# Incident psoriasis in atopic dermatitis: a large-scale cohort study of disease- and treatment-associated risks

**DOI:** 10.3389/fimmu.2026.1900942

**Published:** 2026-08-28

**Authors:** Florian Thaqi, Katja Bieber, Hatim Kerniss, Khalaf Kridin, Philip Curman, Ralf J. Ludwig

**Affiliations:** 1 Luebeck Institute of Experimental Dermatology, University of Luebeck, Luebeck, Germany; 2 Department of Cardiology, Goethe University Hospital, Frankfurt am Main, Germany; 3 Azrieli Faculty of Medicine, Bar-Ilan University, Safed, Israel; 4 Unit of Dermatology and Skin Research Laboratory, Barch Padeh Medical Center, Poriya, Israel; 5 Dermato-Venereology Clinic, Karolinska University Hospital, Stockholm, Sweden; 6 Department of Medical Epidemiology and Biostatistics, Karolinska Institutet, Stockholm, Sweden; 7 Dermatology and Venereology Division, Department of Medicine (Solna), Karolinska Institutet, Stockholm, Sweden; 8 Institute and Comprehensive Center for Inflammation Medicine, University of Luebeck, Luebeck, Germany

**Keywords:** atopic dermatitis, cohort study, dupilumab, pharmacoepidemiology, psoriasis

## Abstract

**Background:**

Clinical and genetic evidence on the association between atopic dermatitis (AD) and subsequent psoriasis remains conflicting, and it is unclear whether this risk is modified by systemic treatments. Recent reports suggest that type 2-targeted biologics may unmask psoriasis in patients with AD, but data are limited. We thus aimed to assess whether AD is associated with incident psoriasis and whether this risk differs by systemic treatment, particularly biologics versus conventional systemic immunosuppressants (cvIS).

**Methods:**

Scoping analyses informed a locked analytic design, preregistration at Open Science Framework, and confirmatory execution. Propensity score-matched analyses compared AD with non-AD controls and biologics with cvIS. Sensitivity analyses, including multivariable Cox proportional hazards (CPH) analyses, and control outcomes assessed robustness.

**Results:**

Among ~300,000 matched pairs, AD was associated with increased psoriasis risk [primary hazard ratio (HR) 3.81, 95% confidence interval (CI) 3.35–4.34], consistent across all eight sensitivity analyses and CPH. Biologic treatment was associated with reduced psoriasis risk versus cvIS (primary HR 0.20; 95% CI 0.11–0.35), consistent across eight of nine evaluable sensitivity analyses and CPH. Positive and negative control outcomes showed expected directional patterns.

**Conclusions:**

Acknowledging limitations including residual confounding and coding misclassification, AD was associated with increased psoriasis risk and biologics with lower psoriasis risk than cvIS.

## Introduction

Atopic dermatitis (AD) and psoriasis are the two most prevalent chronic inflammatory skin diseases (CISDs), together affecting over 10% of the global population. They have been considered immunologically distinct: AD driven by type 2 helper T (Th2) cell-mediated inflammation with elevated interleukin-4 (IL-4) and IL-13, whereas psoriasis is driven by Th1/Th17 responses with predominant IL-17 and IL-22 production (1, 2). In support of this notion, a key study demonstrated that patients with concurrent AD and psoriasis exhibited distinct T-cell infiltrates in lesions from each disease, with an antagonistic clinical course (3). However, this traditional dichotomy has been challenged by observations of phenotypic overlap, mixed immunophenotypes, and disease transition (4–6).

Epidemiological evidence on the relationship between AD and subsequent psoriasis risk remains conflicting. Population-based cohort studies from Taiwan have reported bidirectional associations, suggesting that patients with either disease may be at elevated risk for developing the other (7). A UK primary care cohort of over 170,000 patients with AD similarly found increased risk of incident psoriatic arthritis, particularly in severe AD (8). In contrast, a German cross-sectional study of 90,000 dermatologist-examined individuals found that type 2 inflammatory diseases, including AD, were significantly less frequent in persons with psoriasis, supporting the traditional mutual exclusivity hypothesis (9). Genetic evidence yields conflicting results. Mendelian randomization studies documented that AD did not affect psoriasis risk (10) or that AD may act as a protective factor for psoriasis (11). Genetic analyses have also shown that AD and psoriasis harbor opposing risk alleles at shared loci, with no evidence of shared loci acting in the same direction, supporting the notion of partial mutual exclusivity between the two diseases (12). This discordance in observational and genetic evidence leaves uncertainty regarding whether AD itself confers psoriasis risk in routine clinical care.

The emergence of type 2-targeted biologics, specifically dupilumab, tralokinumab, and lebrikizumab, has transformed AD management. However, case reports and pharmacovigilance signals have suggested that these biologics, especially dupilumab, may unmask or trigger psoriasis (termed as switch), potentially through Th2-to-Th17 immune skewing (13–15). In an Italian multicenter retrospective cohort study including 5,899 patients with AD, psoriasis developed in 1.3% receiving dupilumab and 2.1% on tralokinumab (14). However, this study lacked a conventional systemic immunosuppressants (cvIS) comparator, precluding direct assessment of whether psoriasis risk differs by treatment class. In line, a recent TriNetX cohort study reported a 58% higher psoriasis risk in adult AD treated with dupilumab as opposed to other systemic agents (16). However, methodological limitations warrant caution: Treatment exposure was not tightly anchored to AD diagnosis (potentially including patients treated for other indications); the analysis was restricted to adults, limiting generalizability to pediatric populations where AD is most prevalent; and some cohort definitions included immortal time bias. Furthermore, preregistration was not reported, limiting assessment of whether analyses were hypothesis-driven versus exploratory (17). As such, comparative data evaluating biologics versus cvIS with rigorous design features remain limited.

Given these knowledge gaps, we conducted a large-scale, preregistered retrospective cohort study to address two aims (1): to determine whether AD is associated with increased incident psoriasis risk compared with matched non-AD controls, and (2) to assess whether psoriasis risk among patients with AD differs according to systemic treatment exposure, particularly type 2-targeted biologics versus cvIS. This preregistered study employed propensity score matching, prespecified sensitivity analyses, Cox proportional hazards (CPH) analyses, and positive and negative control outcomes to strengthen the robustness of observed associations.

## Methods

**Figure 1:**
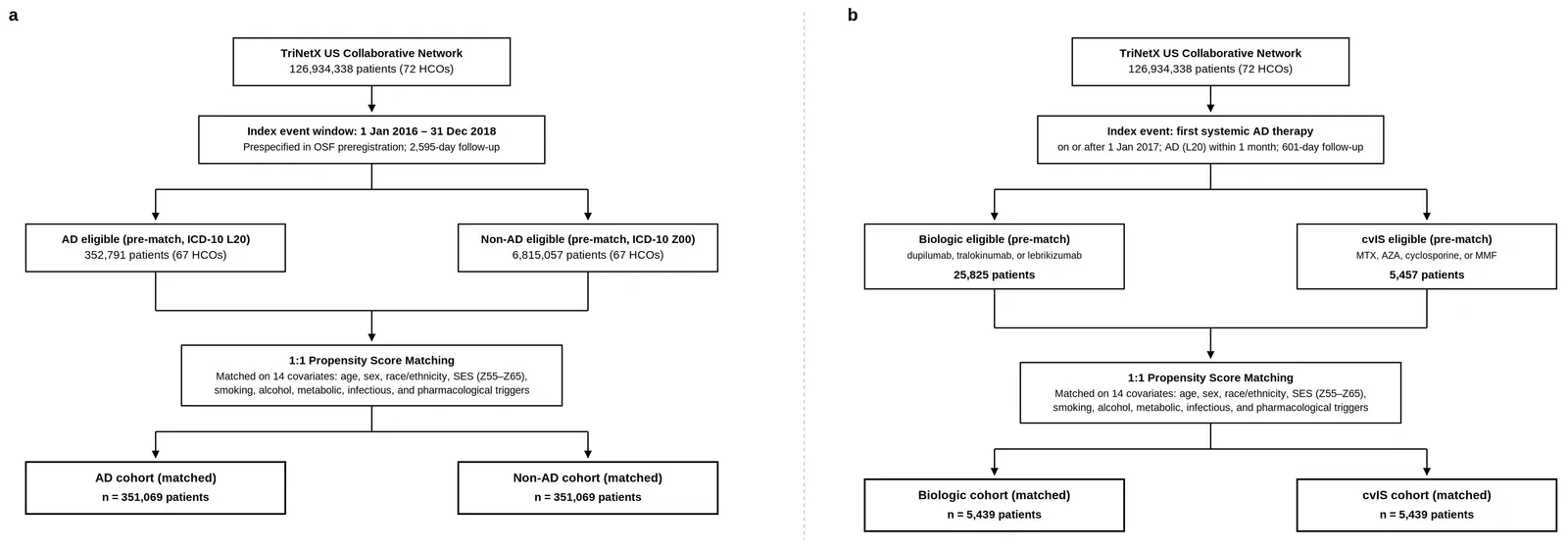
Patient flow diagram. Cohort construction from the TriNetX US Collaborative Network for **(A)** Aim 1, comparing atopic dermatitis (AD; ICD-10-CM L20) with non-AD controls (Z00 encounter), and **(B)** Aim 2, comparing type 2-targeted biologics (dupilumab, tralokinumab, or lebrikizumab) with cvIS (methotrexate, azathioprine, ciclosporin, or mycophenolate mofetil). Both aims were prespecified in an Open Science Framework preregistration prior to data extraction, and cohorts were matched 1:1 on 14 covariates (see Methods). Because the cohort queries have no right-censoring date, re-execution at a later date will return larger cohorts as HCOs upload additional data or new HCOs join the network. HCO, healthcare organization.

This study addressed two aims: Aim 1 assessed whether AD is associated with a changed incident psoriasis risk compared with matched non-AD controls. Aim 2 investigated if psoriasis risk in patients with AD differs by systemic treatment exposure, particularly biologics versus cvIS (**Figure 1**).

### Study design and data source

A propensity score-matched retrospective cohort study was conducted using the US Collaborative Network of the federated electronic health record (EHR) platform TriNetX (following established procedures) (18). The study started with an exploratory scoping phase to assess feasibility [including cohort definitions, the number of covariates accommodated by the propensity score matching (PSM), and sufficiency of outcome events], followed by a design freeze, preregistration [Open Science Framework (https://osf.io/8f2s4/registrations), registered 26 March 2026], and confirmatory execution (performed 27–28 March 2026).

Analyses were performed within the TriNetX platform using federated queries, with only aggregated results returned to investigators. The network was selected for its large EHR volume, detailed covariate documentation, and representation of the US healthcare-seeking population (19, 20). At the time of analysis, 65 of a total of 66 healthcare organizations (HCOs) within the network provided data from over 110 million electronic healthcare records (EHRs). The study evaluated whether AD was associated with an increased risk of incident psoriasis and whether psoriasis risk differed according to treatment exposure among patients with AD. Reporting followed the RECORD (Aim 1) and RECORD-PE (Aim 2) guidelines (21, 22). The study evaluated whether AD was associated with an increased risk of incident psoriasis and whether psoriasis risk differed according to treatment exposure among patients with AD. The analysis used de-identified secondary data and involved no interaction with human participants. Data were de-identified according to §164.514(a) of the HIPAA Privacy Rule. The TriNetX platform operates under a federated governance framework compliant with HIPAA, the General Data Protection Regulation (GDPR), and the Lei Geral de Proteção de Dados (LGPD); therefore, institutional review board approval was not required.

### Preliminary scoping analysis and design finalization

Before formal execution of the preregistered analyses, a scoping analysis was conducted for both study questions using the logic of the planned primary analyses. In this phase, outcomes were counted beginning 1 day after the index event without additional follow-up restrictions to verify technical feasibility of cohort definitions, covariate retrieval, comparator construction, and outcome ascertainment within TriNetX. This step served only to confirm the feasibility and operational validity of the intended design and was not used for confirmatory inference. For Aim 2, the scoping analysis revealed that only the biologic versus cvIS and dupilumab versus cvIS comparisons yielded sufficient sample sizes for comparative analysis; Janus kinase inhibitor (*n* = 983) and IL-13 inhibitor (*n* = 430) cohorts fell below the prespecified threshold of 1,000 patients after cohort construction and were therefore not taken forward. After review of the scoping outputs, the final analytic framework was fixed at a design freeze point (26 March 2026), defined as the transition from exploratory setup to locked confirmatory design. At this point, cohort definitions, covariates, outcomes, comparators, and sensitivity analyses were finalized and no longer modified on the basis of observed data patterns. Once preregistered, the full study was executed.

### Study population and index events

#### Aim 1

Cases were defined as patients with a first recorded diagnosis of AD [International Classification of Diseases, 10th Revision, Clinical Modification (ICD-10-CM) L20] between 1 January 2016 and 31 December 2018. Controls were defined as patients with a first recorded general health examination encounter (Z00) during the same period. For both cohorts, the index date was the date of the first eligible record of the respective index event. At data retrieval, patients with recorded psoriasis, burns, and corrosions of external body surface, appendectomy, presbycusis, topical betamethasone exposure, or prior AD on or before the index date were excluded. Detailed cohort definitions and index event specifications are provided in **Supplementary Table 1**.

#### Aim 2

Treatment cohorts were defined from first eligible systemic treatment exposure on or after 1 January 2017. This design was chosen in part to minimize distortion from treatment-era effects, which may influence comparative estimates when biologics and cvIS are assessed across substantially different prescribing periods. The primary exposed cohort consisted of patients receiving dupilumab, tralokinumab, or lebrikizumab, and the comparator cohort of patients receiving methotrexate, azathioprine, mycophenolate mofetil, or ciclosporin. In both groups, the index date was the date of first eligible treatment exposure. Exposure was defined at first eligible treatment record, with follow-up proceeding irrespective of subsequent treatment changes, consistent with an intention-to-treat (ITT)-like framework. To align treatment exposure with indication, a recorded diagnosis of AD (L20) was required within 1 month on or before treatment initiation. To ensure treatment-naïve comparisons, patients in the biologic cohort with prior exposure to methotrexate, azathioprine, mycophenolate mofetil, ciclosporin, or Janus kinase inhibitors (abrocitinib, upadacitinib, and baricitinib) on or before index were excluded. Likewise, patients in the cvIS cohort with prior exposure to dupilumab, tralokinumab, lebrikizumab, or Janus kinase inhibitors on or before index were excluded. Outcomes recorded on or before the index event were excluded at data retrieval. Detailed cohort definitions and index event specifications are provided in **Supplementary Table 2**.

To contextualize the treatment comparison against temporal prescribing patterns, scoping analyses quantified the number of patients receiving each eligible therapy in 6-month intervals across a 6-year window spanning 3 years before and 4 years after the year in which the respective drug was licensed for AD. Because JAK and IL-13 inhibitor cohorts did not meet the prespecified minimum threshold of 1,000 patients described above, the temporal-prescribing analysis was restricted to dupilumab and cvIS. Cohort definitions otherwise mirrored the primary analysis, but without exclusion of outcomes recorded before index.

### Outcomes

Outcomes were defined by ICD-10-CM, SNOMED, or CPT codes and included psoriasis vulgaris (L40.0), burns and corrosions of external body surface (T20–T25), presbycusis (H91.1), and appendectomy (CPT 1014622, 44950, and 44970, and SNOMED 80146002), which served as negative control outcomes across all analyses. Topical betamethasone (RxNorm 1514; topical product) served as a positive control outcome in Aim 1. Outcomes recorded before the start of follow-up were excluded at data retrieval. If the number of events was too low for individual negative control outcomes, a composite of all was used. Additional outcomes listed in the preregistration (AD, acne vulgaris, rosacea, hidradenitis suppurativa, vitiligo, alopecia areata, lichen planus, and depression) were not analyzed, as these entries were inadvertently carried over into the preregistration and were not part of the prespecified study objective addressed here. This did not affect cohort construction, outcome ascertainment for the reported endpoint, or the analyses presented in this manuscript. As a *post-hoc* analysis in response to peer review, drug exposure patterns were characterized descriptively in both treatment arms (Aim 2). The number of subsequent dispensing events was quantified within the observation window, and follow-up durations were summarized as median [interquartile range (IQR)]. Patient counts differ slightly from the primary Aim 2 cohort because these descriptive analyses were performed on data extracted after the primary analysis, reflecting continued expansion of the network.

### Covariates and propensity score matching

#### Aim 1

Covariates for PSM were selected *a priori* based on clinical relevance and scoping feasibility. Because the scoping analysis showed that no more than 14 covariates could be accommodated in the TriNetX matching framework, the final propensity score model included age at index, female sex, White ancestry, Black or African American ancestry, socioeconomic and psychosocial circumstances (Z55–Z65), personal history of nicotine dependence (Z87.891), acute pharyngitis (J02), nicotine dependence (F17), alcohol-related disorders (F10), diabetes mellitus (E08–E13), overweight and obesity (E66), beta blockers/related (VA CV100), ACE inhibitors (VA CV800), and non-steroidal anti-inflammatory analgesics (VA CN104), all assessed up to 1 day before index. Propensity scores were estimated by logistic regression and cohorts matched 1:1 using greedy nearest-neighbor matching with a caliper of 0.1 pooled standard deviations of the logit of the propensity score; balance was assessed using standardized mean differences. Additional retrieved but unmatched covariates included male sex, Hispanic or Latino ancestry, Asian ancestry, family history of skin and subcutaneous tissue disease (Z84.0), reaction to severe stress and adjustment disorders (F43), Crohn’s disease (K50), ulcerative colitis (K51), HIV disease (B20–B24), lithium salts (VA CN750), and antimalarials (VA AP101). Based on the scoping analysis, follow-up was fixed at 2,595 days.

#### Aim 2

PSM for Aim 2 followed the same approach as Aim 1, with all covariates included. Follow-up was fixed at 601 days based on the scoping analysis.

### Sensitivity analyses

#### Aim 1

The primary analysis compared incident AD with non-AD controls. Follow-up began 1 day after the index event and was restricted to the maximum common fixed follow-up period supported by both cohorts, as determined in the scoping analysis (2,595 days). Thus, the prespecified follow-up window corresponded to the shorter of the two cohort-specific available follow-up durations identified during scoping and was applied uniformly to both cohorts and across all analyses. Prespecified sensitivity analyses included a 3-month lag-time analysis (S1) to address reverse causation, restriction to patients with at least 6 months of baseline observation (S2) to reduce prevalent-user bias, and a stricter AD definition requiring two recorded diagnoses of AD (L20) to reduce single-diagnosis misclassification, with the first instance of AD occurring within 6 weeks to 12 months before any instance of AD (Group 1A); the index date remained the Group 1A event, so this definition did not introduce immortal time bias (S3). Alternatively, in S4, as a literature-comparability check, AD was defined using an alternative two-diagnosis logic requiring a second qualifying L20 diagnosis within 1 month on or after the first AD diagnosis; the corresponding logic was applied to controls. This design introduces immortal time bias because cohort qualification depends on a post-index event but was included for comparison with commonly used observational database definitions. Because of computational constraints related to cohort size, PSM in S4 was limited to 13 covariates (excluding non-steroidal anti-inflammatory analgesics). In S5, to exclude pre-existing inflammatory dermatoses that could confound outcome ascertainment, diagnoses of dermatitis and eczema (L20–L30), papulosquamous disorders (L40–L45), and other disorders of the skin and subcutaneous tissue (L49–L50) recorded before index were excluded in both cases and controls, except for L20 in the AD group. In S6, as an active-comparator check to partially adjust for dermatology-seeking behavior, incident acne vulgaris served as an active comparator cohort. Additional analyses were stratified by age, to test age-specific effect modification, separately including patients aged under 18 years (S7) and 18 years or older (S8). Multivariable CPH (S9) prespecified as a robustness check against KM proportionality assumption violations could not be executed due to computational constraints; the model failed to converge even when covariates were reduced to six. This was acknowledged in the preregistration as potentially problematic. As a *post-hoc* alternative, the non-AD control definition was modified by replacing general health examination encounters (Z00) with acne vulgaris (L70.0) as the index event.

#### Aim 2

The primary analysis compared treatment cohorts using follow-up beginning 1 day after the index event and uniformly capped at the maximum common follow-up duration available in both cohorts (601 days), corresponding to the shorter observable follow-up of either cohort identified during scoping. Prespecified sensitivity analyses included a 3-month lag-time analysis (S1) to address early post-treatment reverse causation, restriction to patients with at least 6 months of baseline observation before index (S2) to reduce prevalent-user bias, and a stricter AD definition to reduce misclassification, requiring two recorded diagnoses of AD (L20), with cohort entry anchored to the qualifying index event so that no immortal time bias was introduced (S3). In S4, the same alternative two-diagnosis AD definition as in Aim 1 was applied in both treatment groups, acknowledging the same immortal time bias considerations described above. In S5, to exclude pre-existing inflammatory dermatoses, the same exclusion of pre-index diagnoses as in Aim 1 was applied in both treatment groups. Additional analyses were stratified by age, separately including patients aged under 18 years (S6) and 18 years or older (S7). In S8, to isolate the largest single biologic from the pooled comparison, dupilumab alone was compared with cvIS. JAK inhibitor comparison was prespecified but not performed as the cohort did not meet the minimum threshold of 1,000 patients. In S9, to test whether anchoring on treatment rather than AD diagnosis altered the finding, exclusion of outcomes prior to the index event was temporally anchored on applied treatments, as opposed to AD diagnosis. Three additional *post-hoc* sensitivity analyses were added in response to peer review to evaluate whether the primary finding held across distinct phases of biologic uptake in the network. In S10, treatment cohorts were restricted to first systemic treatment between January 2017 and December 2020, corresponding to the period during which cvIS remained numerically dominant. In S11, treatment cohorts were restricted to first systemic treatment from January 2021 onwards, corresponding to the period after biologic prescribing had exceeded that of cvIS. Both *post-hoc* analyses used the same cohort definitions, PSM procedure, and analytic framework as the primary Aim 2 analysis. In another *post-hoc* analysis, the cvIS cohort was restricted to MTX (with exclusion of other cvIS agents, biologics, and JAK inhibitors) to address concerns regarding heterogeneity of the pooled cvIS comparator. Because of low output count, this analysis was performed using multivariate CPH. In S12 (again preregistered), an additional sensitivity analysis was carried out using multivariable CPH (see below). Because contributing HCOs change over time and cohort queries have no right-censoring date (Aim 2), cohort sizes may differ modestly between the primary and sensitivity analyses, reflecting continued data accrual in the network.

### Multivariable Cox proportional hazards analyses

An additional sensitivity analysis was performed using multivariable CPH. For both aims, the covariate set mirrored that used in Aim 2 PSM, with female sex replaced by male sex as the reference category (TriNetX platform default). Aim 1: AD status was entered as the main predictor. Follow-up was fixed at 2,595 days, consistent with the primary Kaplan–Meier analysis. Outcomes included psoriasis vulgaris, topical betamethasone (positive control), and one negative control outcome (presbycusis). As anticipated in the preregistration, computational constraints prevented Cox model execution using the original non-AD control definition (Z00). As a *post-hoc* alternative, the control cohort was redefined using acne vulgaris (L70.0) as the index event. Aim 2: Treatment cohort (biologics versus cvIS) was entered as the main predictor. Follow-up was fixed at 601 days, consistent with the primary Kaplan–Meier analysis. Outcomes included psoriasis vulgaris and a composite negative control outcome (burns and corrosions, presbycusis, and appendectomy), as individual negative control outcomes had insufficient event counts for separate analysis.

### Missing data handling

Missing covariate values were retained in all analyses. In PSM, missingness was treated as a separate category. In multivariable CPH models, missing values were likewise retained.

### Statistical analysis

Baseline characteristics were summarized before and after PSM. Continuous variables were compared using *t*-tests and binary or categorical variables using *z*-tests; covariate balance was assessed using standardized mean differences. Time-to-event analyses were performed using the Kaplan–Meier method and compared using the log-rank test, with hazard ratios (HRs) and 95% confidence intervals (CIs) reported For Kaplan–Meier analyses, TriNetX reports a proportionality test including a chi-square statistic and *p*-value. Consistent with platform guidance, these were interpreted qualitatively rather than as strict inferential thresholds: larger chi-square values were taken to indicate less proportionality and smaller values were taken to indicate greater proportionality. Bonferroni correction was applied for multiple testing across prespecified outcomes [Aim 1: α(adjust) = 0.01; Aim 2: α(adjust) = 0.025]. *E*-values were computed for the point estimate and for the 95% confidence limit nearest the null by direct substitution of the HR for the risk ratio (23).

## Results

The results are presented in two parts: Aim 1 examines the association between AD and subsequent psoriasis risk, whereas Aim 2 evaluates psoriasis risk according to systemic treatment exposure in AD.

### Risk of subsequent psoriasis in patients with AD

In the primary analysis, 351,070 patients with AD and 6,888,769 non-AD controls were identified before matching. PSM yielded 351,069 matched pairs. Before matching, cohorts differed substantially; after matching, all baseline covariates were well balanced (SMDs <0.10). Median follow-up was 2,595 days in both cohorts [IQR: 1,219 (AD) and 1,129 days (controls)]. Full baseline characteristics are presented in **Supplementary Table 3**.

**Figure 2:**
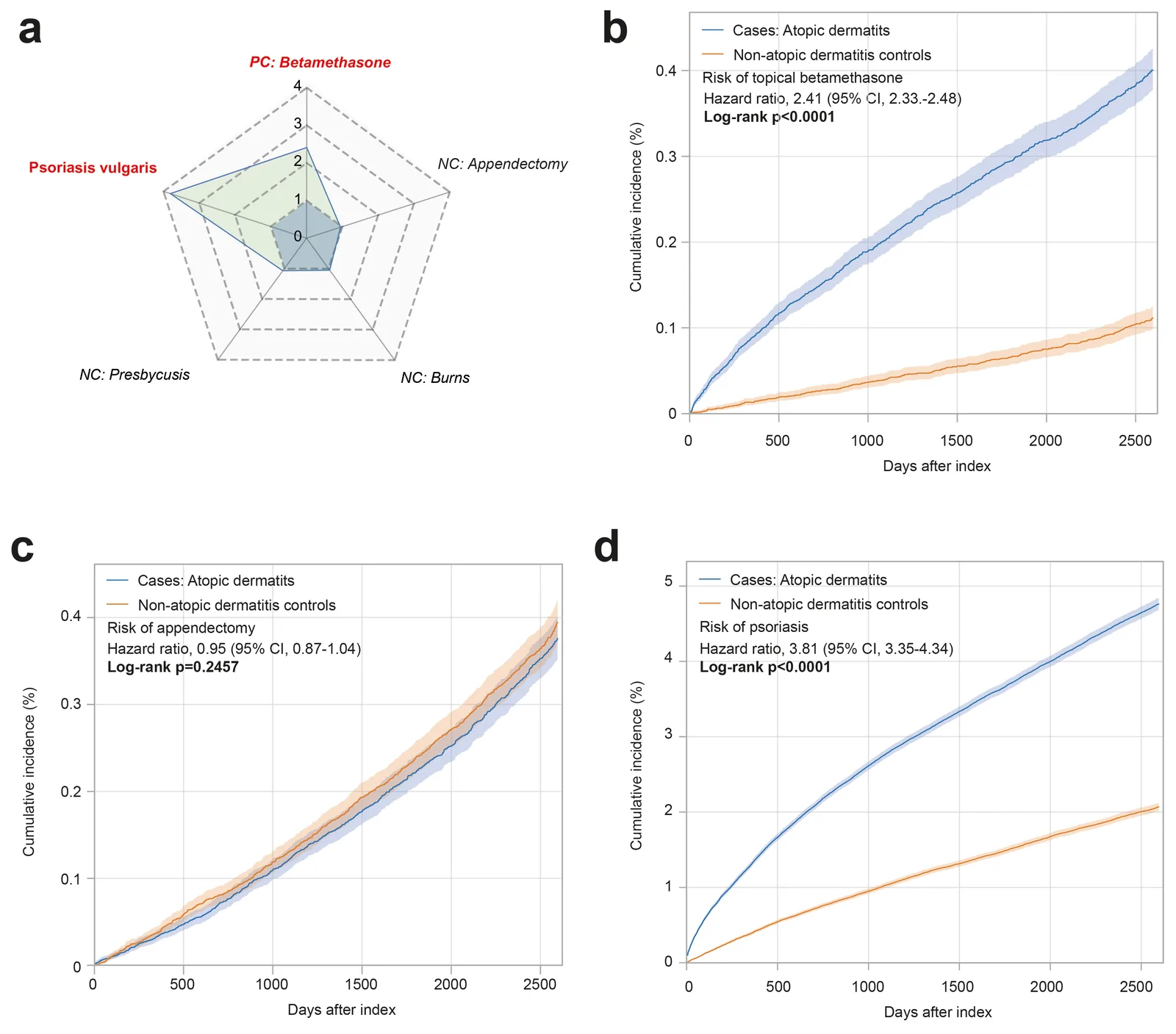
Primary outcome and control outcome analyses in patients with atopic dermatitis versus matched non-atopic dermatitis controls. **(A)** Radar plot depicting hazard ratios (HRs) from the primary analysis across all evaluated endpoints. Positive control (PC) and negative control (NC) outcomes are indicated in italics; red labels denote statistically significant associations. **(B–D)** Kaplan–Meier curves showing cumulative incidence of **(B)** topical betamethasone exposure (positive control), **(C)** appendectomy (negative control), and **(D)** psoriasis vulgaris (primary outcome) in patients with AD and matched non-AD controls. Shaded bands represent 95% confidence intervals.

Psoriasis was documented in 1,088 of 351,069 patients with AD (0.31%) versus 290 of 351,069 matched controls (0.08%). Kaplan–Meier analysis demonstrated higher cumulative incidence in the AD cohort (*p* < 0.0001, HR 3.81; 95% CI, 3.35–4.34, **Figure 2**; **Supplementary Table 3**). AD was associated with an increased risk of subsequent psoriasis. Comparator choice influenced the magnitude of this association. In sensitivity analyses using Z00 controls, HRs ranged from 3.07 to 4.04, whereas substituting acne vulgaris as an active comparator yielded a more conservative estimate (HR 1.73; 95% CI, 1.56–1.93), and CPH using the acne comparator similarly attenuated the association (HR 1.39; 95% CI, 1.27–1.52, *p* < 0.001, **Table 1**). This attenuation likely reflects partial adjustment for healthcare-seeking behavior and dermatology-specific coding patterns shared between AD and acne patients. Notwithstanding this comparator-dependent variation, the direction of association was consistent across all prespecified sensitivity analyses (HR range: 1.39–4.04, **Supplementary Table 3**). The association also persisted after applying a 3-month lag time (HR 3.61; 95% CI, 3.16–4.12), restricting to patients with at least 6 months of baseline data (HR 3.48; 95% CI, 3.03–3.99), using stricter AD definitions with and without potential immortal time bias (HR 4.04; 95% CI, 3.20–5.12 and HR 3.41; 95% CI, 3.01–3.86, respectively), and excluding patients with pre-existing inflammatory skin diseases (HR 3.07; 95% CI, 2.53–3.73). The association was observed in both age strata: patients younger than 18 years (HR 2.86; 95% CI, 2.28–3.59) and those aged 18 years or older (HR 3.41; 95% CI, 2.95–3.94).

**Table 1:** Multivariable Cox proportional hazards model for incident psoriasis vulgaris in patients with atopic dermatitis versus acne controls.

Covariate	Hazard ratio	Lower 95% CI	Upper 95% CI	*p*-value
AD vs. acne	1.39	1.27	1.52	<0.001
Age at index (per year)	1.03	1.03	1.03	<0.001
Male sex	0.95	0.86	1.05	0.298
Healthcare utilization (visits)	0.92	0.78	1.09	0.323
Hispanic or Latino	0.72	0.61	0.86	<0.001
White	0.82	0.71	0.93	0.003
Black or African American	0.34	0.29	0.42	<0.001
Asian	1.01	0.83	1.24	0.89
Persons with potential health hazards related to socioeconomic and psychosocial circumstances (Z55–Z65)	0.98	0.75	1.29	0.898
Family history of diseases of the skin and subcutaneous tissue (Z84.0)	1.52	0.57	4.05	0.406
Personal history of nicotine dependence (Z87.891)	1.02	0.82	1.25	0.892
Acute pharyngitis (J02)	1	0.88	1.14	0.969
Nicotine dependence (F17)	1.37	1.14	1.65	<0.001
Alcohol-related disorders (F10)	1.08	0.8	1.47	0.607
Reaction to severe stress and adjustment disorders (F43)	0.99	0.81	1.22	0.949
Diabetes mellitus (E08–E13)	1.01	0.84	1.21	0.906
Overweight/obesity (E66)	1.36	1.19	1.56	<0.001
Crohn’s disease (K50)	3.4	2.42	4.79	<0.001
Ulcerative colitis (K51)	1.72	1.11	2.64	0.014
Human immunodeficiency virus (HIV) disease (B20)	0.94	0.42	2.11	0.887
Lithium salts (CN750)	2.25	1.34	3.77	0.002
Beta blockers/related (CV100)	1.15	0.99	1.33	0.074
ACE inhibitors (CV800)	0.82	0.68	0.98	0.029
Non-steroidal anti-inflammatory analgesics (CN104)	1.14	1.01	1.28	0.033
Antimalarials (P01B)	1.44	1.07	1.94	0.016

Shown are results from a multivariable Cox proportional hazards regression model assessing incident psoriasis vulgaris in patients with atopic dermatitis compared with acne controls. The model included 351,074 patients with atopic dermatitis and 334,717 patients with acne and used a follow-up period of 2,595 days. Estimates are presented as hazard ratios with 95% confidence intervals. The model was adjusted for the covariates shown in the table.

Control outcome analyses supported the analytic framework. The positive control (topical betamethasone) was consistently elevated in patients with AD across the primary analysis, sensitivity analyses, and CPH. Among negative controls, appendectomy remained null throughout Kaplan–Meier analyses. Presbycusis was null in the primary analysis, most sensitivity analyses, and CPH, but showed an association in the acne comparator analysis. Burns and corrosions showed only small, inconsistent associations (**Supplementary Tables 3**, **4**).

### Psoriasis risk in AD by systemic treatment exposure

In the primary analysis, 25,825 patients receiving biologic therapy and 5,457 receiving cvIS were identified before matching. PSM yielded 5,439 matched pairs. Before matching, cohorts differed modestly; after matching, all baseline covariates were well balanced (SMDs <0.10). Median follow-up was 596 days (biologic) and 601 days (cvIS), with IQRs of 394 and 12 days, respectively. Full baseline characteristics are presented in **Supplementary Table 5**.

In a *post-hoc* descriptive analysis of drug exposure patterns in the matched cohorts, within-class persistence was essentially equivalent between arms: 68.6% (4,358/6,357) of biologic-treated patients and 64.6% (4,108/6,357) of cvIS-treated patients had at least one within-class dispensing captured during follow-up, with a median of 3 subsequent dispensings in each arm (biologic: mean 4.11, SD 4.95; cvIS: mean 4.92, SD 12.40; *p* = 0.35 by *t*-test for within-class dispensings).

**Figure 3:**
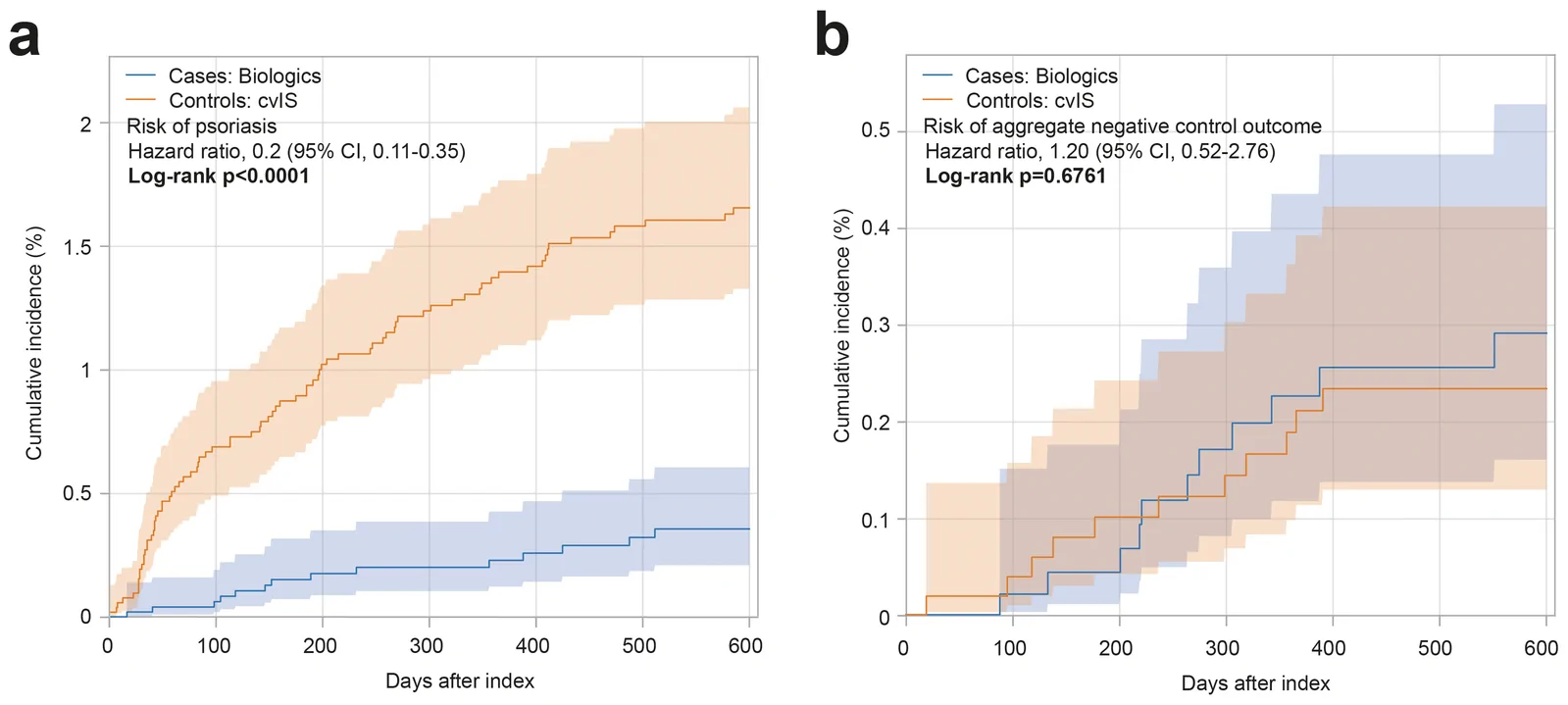
Kaplan–Meier analyses of psoriasis vulgaris and aggregate negative control outcomes in patients with atopic dermatitis (AD) treated with biologics versus conventional systemic immunosuppressants (cvIS). **(A)** Cumulative incidence of psoriasis vulgaris. Biologic therapy was associated with a lower cumulative incidence of psoriasis vulgaris than cvIS (HR, 0.20; 95% CI, 0.11–0.35; log-rank *p* < 0.0001). **(B)** Cumulative incidence of the aggregate negative control outcome. No association was observed between treatment group and the aggregate negative control outcome (HR, 1.20; 95% CI, 0.52–2.76; log-rank *p* = 0.6761). Shaded areas indicate 95% confidence intervals. Curves are displayed according to Kaplan–Meier convention, with the last observed survival probability carried forward until the next event or censoring.

Psoriasis was documented in 14 of 5,424 patients receiving biologic therapy (0.26%) versus 79 of 5,352 matched patients receiving cvIS (1.48%). Kaplan–Meier analysis demonstrated lower cumulative incidence in the biologic cohort (*p* < 0.0001, HR, 0.20; 95% CI, 0.11–0.35; **Figure 3**; **Supplementary Table 5**).

The primary finding was directionally consistent across all evaluable sensitivity analyses, with HRs consistently below 1.0 (range: 0.20–0.59, **Supplementary Table 5**). The association persisted after restricting to patients with at least 6 months of baseline data (HR 0.32; 95% CI, 0.18–0.57), using stricter AD definitions (HR 0.33; 95% CI, 0.19–0.59, and HR 0.36; 95% CI, 0.23–0.56), among patients aged 18 years or older (HR 0.33; 95% CI, 0.20–0.53), for dupilumab versus cvIS specifically (HR 0.40; 95% CI, 0.26–0.61), and when the index event was temporally anchored on treatment initiation (HR 0.35; 95% CI, 0.22–0.54). After applying a 3-month lag time, the association remained directionally consistent but was only nominally significant (HR 0.59; 95% CI, 0.36–0.96; *p* = 0.032). In two *post-hoc* temporal sensitivity analyses added to address era-related selection concerns raised in peer review, the association was preserved in both the cvIS-dominant phase of the study period (2017–2020: HR 0.34; 95% CI 0.19–0.62; S10) and the biologic-dominant phase (2021 onwards: HR 0.21; 95% CI 0.11–0.40; S11). In a further *post-hoc* analysis prompted by peer review, restricting the cvIS cohort to MTX (to address concerns regarding comparator class heterogeneity), event counts were below the TriNetX display threshold for Kaplan–Meier analysis, but multivariable Cox regression yielded an HR of 0.21 (95% CI 0.16–0.29; *p* < 0.0001). Analyses excluding pre-existing inflammatory skin diseases and those restricted to patients younger than 18 years were not evaluable due to insufficient outcome events. Multivariable CPH confirmed the association (HR 0.28; 95% CI, 0.22–0.37, *p* < 0.001, **Table 2**).

**Table 2:** Multivariable Cox proportional hazards model for incident psoriasis vulgaris in patients treated with biologics versus conventional systemic immunosuppressants.

Covariate	Hazard ratio	Lower 95% CI	Upper 95% CI	*p*-value
Biologic vs. cvIS	0.28	0.22	0.37	<0.001
Age at index (per year)	1.02	1.01	1.02	<0.001
Male sex	0.86	0.66	1.13	0.276
Healthcare utilization (visits)	1.26	0.52	3.09	0.611
Hispanic or Latino	0.94	0.55	1.6	0.813
White	1.11	0.74	1.66	0.616
Black or African American	0.36	0.2	0.66	0.001
Asian	1.76	1.09	2.84	0.021
Persons with potential health hazards related to socioeconomic and psychosocial circumstances (Z55–Z65)	0.88	0.45	1.71	0.695
Family history of diseases of the skin and subcutaneous tissue (Z84.0)	0.66	0.16	2.69	0.562
Personal history of nicotine dependence (Z87.891)	1.17	0.76	1.8	0.485
Acute pharyngitis (J02)	0.84	0.54	1.3	0.439
Nicotine dependence (F17)	1.63	1.07	2.51	0.025
Alcohol-related disorders (F10)	1.31	0.68	2.51	0.415
Reaction to severe stress and adjustment disorders (F43)	1.19	0.72	1.97	0.489
Diabetes mellitus (E08–E13)	1.08	0.69	1.69	0.737
Overweight/obesity (E66)	1.45	1	2.1	0.047
Crohn’s disease (K50)	1.17	0.28	4.84	0.827
Ulcerative colitis (K51)	0.5	0.07	3.7	0.498
Human immunodeficiency virus (HIV) disease (B20)	0.86	0.12	6.24	0.881
Lithium salts (CN750)	1.03	0.14	7.54	0.974
Beta blockers/related (CV100)	0.96	0.65	1.42	0.835
ACE inhibitors (CV800)	0.91	0.58	1.43	0.677
Non-steroidal anti-inflammatory analgesics (CN104)	1.16	0.84	1.6	0.36
Antimalarials (P01B)	0.14	0.02	1.03	0.054

Shown are results from a multivariable Cox proportional hazards regression model evaluating incident psoriasis vulgaris in patients treated with biologics compared with those treated with conventional systemic immunosuppressants (cvIS). The analysis included 27,407 patients receiving biologics and 5,940 receiving cvIS, with a follow-up of 601 days. Hazard ratios are presented with 95% confidence intervals. The model was adjusted for the covariates shown in the table.

Negative control outcome analyses supported the analytic framework. In Kaplan–Meier analyses, the composite negative control outcome was evaluable only in the primary analysis and the sensitivity analysis with temporally anchored treatment exposure—both yielded null findings. The remaining sensitivity analyses were not evaluable due to insufficient outcome events (**Supplementary Table 5**). Multivariable CPH was consistent (HR 1.01; 95% CI, 0.57–1.79; *p* = 0.97; **Supplementary Table 6**).

## Discussion

This large-scale, preregistered retrospective cohort study addressed two distinct questions. First, whether AD itself is associated with an altered psoriasis risk, and second, whether the choice of systemic treatment modifies that risk. Regarding the first question, AD was associated with a 1.4- to 4-fold increased risk of subsequent psoriasis compared with matched non-AD controls. Regarding the second question, among patients with AD receiving systemic therapy, biologic treatment was associated with a 65%–80% lower psoriasis risk compared with cvIS.

Relating to the second study question, whether treatment modifies psoriasis risk, the findings run counter to the prevailing concern that type 2-targeted biologics trigger psoriasis. Prior epidemiological studies have reported conflicting results, with some suggesting bidirectional associations and others supporting mutual exclusivity (7–9). The present study, including over 300,000 matched pairs with extended follow-up, provides robust evidence that AD is associated with an increased psoriasis risk in routine clinical care.

The finding that biologic therapy was associated with reduced psoriasis risk compared with cvIS contrasts with recent reports suggesting that type 2-targeted biologics may unmask or trigger psoriasis (13–15). Furthermore, a recent large-scale retrospective cohort study reported a 58% increased psoriasis risk with dupilumab compared with other systemic agents (16). However, the study design may have been susceptible to bias, as it was not preregistered, did not tightly anchor treatment initiation to AD diagnosis, and was restricted to adults. An Italian study had also documented psoriasis in 1.3% of dupilumab-treated and 2.1% of tralokinumab-treated patients with AD in a multicenter retrospective cohort explicitly designed to capture psoriasis outcomes (14), potentially explaining the higher incidence compared with 0.26% observed here. Importantly, neither study included a cvIS comparator, precluding direct assessment of whether psoriasis risk differs by treatment class. The present study addresses this gap and suggests that when biologics are compared with cvIS in a matched framework, biologic therapy is associated with lower, not higher, psoriasis risk.

Notably, the absolute psoriasis incidence differed substantially across cohorts in a pattern potentially consistent with AD severity. In the overall AD versus non-AD comparison, which predominantly captures mild AD managed without systemic therapy, risk for a subsequent psoriasis diagnosis was 0.31%. In contrast, among patients with moderate-to-severe AD requiring systemic treatment, psoriasis incidence was 1.48% in those receiving cvIS—but only 0.26% in those receiving biologics. This gradient suggests that psoriasis risk may scale with AD severity—as observed for cutaneous T cell lymphoma risk in AD (24). If confirmed, this would imply that psoriasis emerging during AD treatment reflects disease-associated risk rather than a treatment-specific effect, with biologic exposure being associated with lower rather than higher risk.

**Figure 4:**
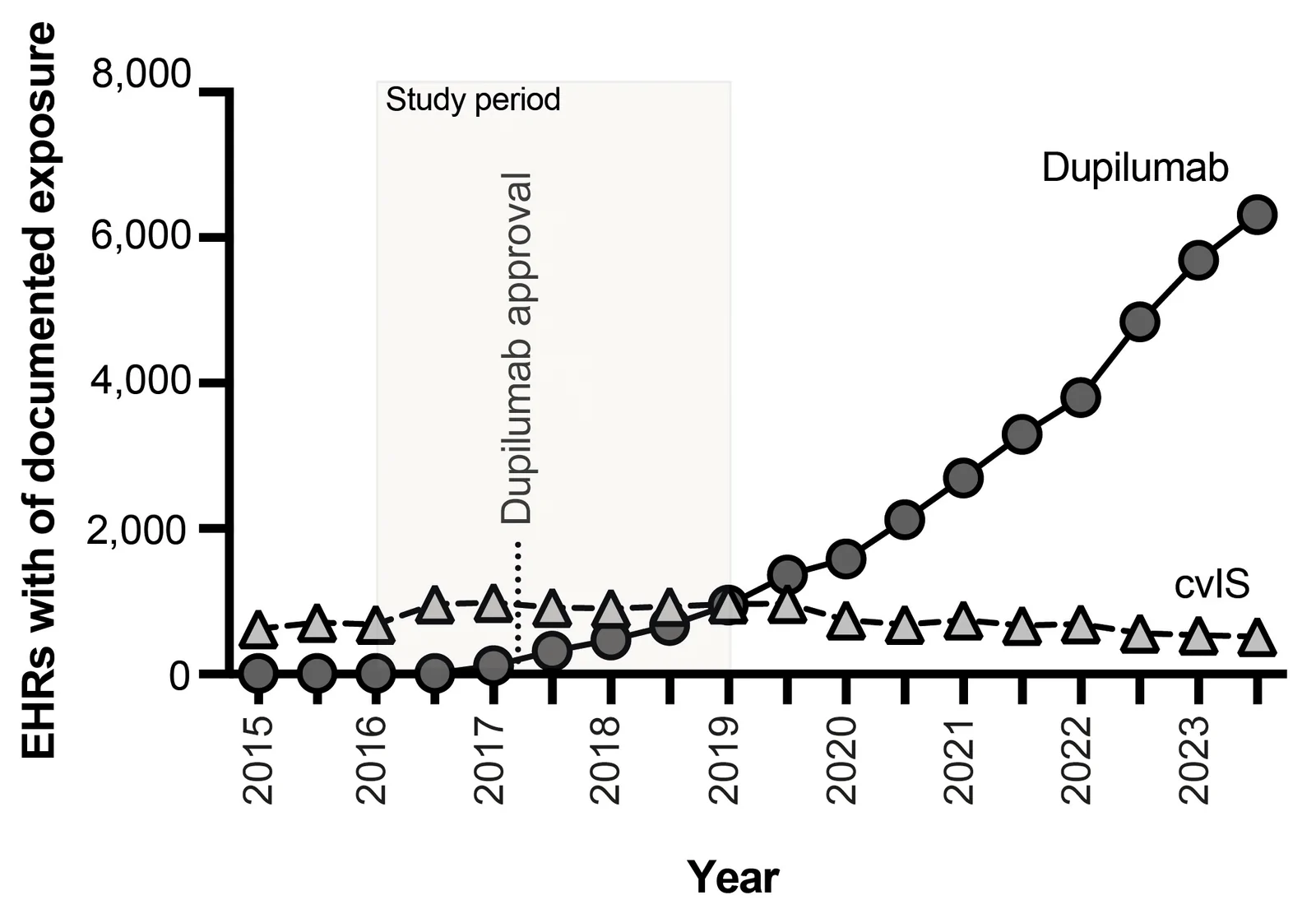
Temporal prescribing patterns of systemic therapies for atopic dermatitis in the TriNetX network. The number of patients with documented exposure to dupilumab and conventional systemic immunosuppressants (cvIS) was quantified in 6-month intervals across a 7-year window spanning 3 years before and 4 years after dupilumab approval for AD. Although the primary treatment analysis included tralokinumab and lebrikizumab alongside dupilumab in the biologic cohort, prescribing numbers for these agents were negligible during the study period; dupilumab therefore serves as the representative biologic agent in this figure. Based on the prespecified threshold for feasible comparative analyses, only dupilumab and cvIS showed sufficient temporal overlap and cohort size for confirmatory comparison. Cohort definitions otherwise mirrored the primary treatment analysis but did not exclude outcomes recorded before index. The shaded area indicates the study period used for the confirmatory treatment comparison, and the dotted vertical line marks the time when the first biologic (dupilumab) was approved for AD.

Several design features support the validity of these findings. Control outcome analyses behaved largely as expected: the positive control (topical betamethasone) was consistently elevated in patients with AD, while negative controls (appendectomy, presbycusis, burns/corrosions) were predominantly null. The study employed a stepwise approach to enhance transparency and minimize analytic flexibility: exploratory scoping analyses informed feasibility, followed by a formal design freeze, preregistration on the Open Science Framework, and confirmatory execution. This framework, herein termed “design freeze”, represents an adaptation of preregistration principles to federated EHR platforms where preliminary feasibility assessment is operationally necessary (17). Additionally, treatment cohorts were temporally anchored to mitigate era effects: eligibility began January 2016, approximately 1 year before dupilumab’s FDA approval for AD (March 2017). This design was necessary because prescribing patterns shifted substantially following biologic approval, which would otherwise limit temporal overlap between treatment groups and compromise comparability. Scoping analyses confirmed sufficient overlap in prescribing patterns during the study period (**Figure 4**).

This study has several limitations inherent to retrospective EHR-based research. Residual confounding cannot be excluded despite PSM for multiple covariates; unmeasured factors such as AD severity, disease duration, or prior treatment history may influence both treatment selection and psoriasis risk. Specifically, TriNetX does not capture disease severity. This constraint is common to federated EHR-based cohorts and represents an important limitation of Aim 2. To partially mitigate this, cohort inclusion in Aim 2 was itself conditional on receipt of systemic AD therapy though this does not fully equalize baseline severity between treatment groups, particularly where line of therapy may differ. Coding misclassification is possible, as diagnoses were ascertained from ICD-10-CM codes without clinical adjudication. Uncertainty regarding the exact treatment indication, including the possibility of protopathic bias, in which cvIS may have been prescribed for early or subclinical psoriasis manifestations subsequently formally diagnosed, cannot be fully resolved, although requiring an AD diagnosis within 1 month of treatment initiation was intended to mitigate this concern. A related limitation is that PSM introduces selection bias whose magnitude depends on covariate specification. In a *post-hoc* sensitivity analysis extending the propensity score model to 29 covariates including atopic-march comorbidities, upper-airway type 2 diseases, inflammatory bowel disease, and inflammatory polyarthropathies, a 5%–10% reduction in matched cohort size relative to the primary analysis was accompanied by an attenuation of the effect estimate from HR 0.20 (95% CI, 0.11–0.35) to HR 0.33 (95% CI, 0.21–0.52), representing an approximate 50% relative change with preserved direction and significance. The matched estimate should therefore be interpreted as applying to the population for whom comparable counterparts existed in both treatment arms. To quantify the residual scope for unmeasured confounding, *E*-values were calculated for all analyses (**Supplementary Tables 3**, **5**). For the primary Aim 2 estimate, these were 9.47 for the point estimate and 5.16 for the confidence limit nearest the null, with point-estimate values ranging from 2.78 to 9.47 across specifications and the 3-month lag-time analysis the least robust. The magnitude of these *E*-values indicates that only substantial unmeasured confounding could account for the observed difference, although they bound the confounding required rather than establishing its absence.

Additionally, the classic phenotype captured by L40.0 may not represent the complete spectrum of AD-to-psoriasis phenotype transitions, as intermediate or overlap psoriasiform presentations may be coded under other dermatitis entities (25). The observed psoriasis incidence of 0.08% among non-AD controls over 2,595 days corresponds to approximately 11.3 cases per 100,000 person-years, which is lower than expected population-based rates in children (approximately 33–41 per 100,000 person-years) (26) and lower than the age-appropriate US adult plaque psoriasis prevalence in the same age band (approximately 60–80 per 100,000 person-years) (27, 28). This discrepancy likely reflects a combination of factors: incomplete capture of diagnoses made outside the contributing healthcare network; the selective nature of the matched non-AD control cohort, which, by design, attenuates the prevalence of many psoriasis-associated risk factors (including obesity, nicotine dependence, and socioeconomic factors) present in the general population; and the anchoring of controls on a Z00 general health examination, which represents a population engaged with preventive care rather than a general demographic sample. Importantly, this ascertainment constraint applies equally to both cohorts and therefore does not compromise the relative comparison that constitutes the primary finding. Furthermore, the ITT-like framework, while reducing selection bias from treatment switching, may not fully capture treatment trajectories including discontinuation, dose changes, or sequential therapy. Proportionality assumptions were occasionally violated in Kaplan–Meier analyses. However, CPH yielded directionally consistent findings, supporting the robustness of the primary conclusions. Additionally, although treatment cohorts were temporally anchored to overlap prescribing periods, residual era effects cannot be fully excluded; biologics and cvIS were still predominantly prescribed in different calendar periods, which may have introduced unmeasured confounding related to evolving clinical practice, patient selection, or concomitant treatment patterns. To partially address this, we conducted two *post-hoc* temporal sensitivity analyses (S10 and S11) partitioning the study period into the phase during which cvIS remained numerically dominant (2017–2020) and the subsequent biologic-dominant phase (2021 onwards). The observed association between biologic exposure and lower psoriasis risk was preserved across both eras, arguing against era-specific selection dynamics as the driver of the primary finding. A further limitation is that the TriNetX platform architecture does not permit combined diagnosis-plus-medication criteria for outcome ascertainment. While psoriasis case definition using L40.0 diagnosis alone has been used in prior TriNetX studies, the specificity of the outcome definition could not be further refined through combined criteria as would be possible for cohort definition. Finally, as an observational study, causal inference is not possible; the observed associations may reflect disease severity, healthcare utilization patterns, or other factors rather than direct biological effects of AD or its treatments on psoriasis development.

The finding that biologics are associated with a lower, rather than higher, psoriasis risk in AD directly contrasts with the recent TriNetX-based analysis, which reported a 58% higher psoriasis risk with dupilumab versus other systemic agents (16). Reconciling these divergent findings requires balanced consideration of both bias-driven and genuine-effect interpretations. On one hand, residual confounding by indication, channeling bias, protopathic bias, and unmeasured AD severity cannot be fully excluded—biases that could theoretically show an apparent protective association in the absence of a genuine treatment effect. On the other hand, several design features reduce the plausibility of a purely bias-driven explanation: preregistration with a formal design freeze prior to data extraction; a new-user, active-comparator framework structurally robust to classical immortal time bias; consistency across sensitivity analyses (HR range 0.20–0.59), including preservation of the effect across both the cvIS-dominant (2017–2020) and biologic-dominant (2021 onwards) prescribing eras; a nearly identical effect estimate in the methotrexate-only comparison (HR 0.21); and prospectively specified negative control outcomes that yielded null results consistent with a valid analytic framework. Yet, biologically plausible mechanisms exist for both directions: increased psoriasis risk under IL-4/IL-13-targeted treatment could arise from Th2-to-Th17 immune re-polarization following IL-4/IL-13 blockade. By contrast, reduced risk could reflect broader downregulation of the type 2 inflammatory milieu that sustains overlapping psoriasiform features in AD–psoriasis phenotype-transition patients.

A further consideration is that the lower absolute psoriasis incidence observed in the biologic arm compared with prior case series and cohort reports likely reflects, at least in part, differences in analytic framework: prior studies typically report incidence conditional on continued dupilumab exposure of at least 16 weeks, whereas the present ITT-like analysis measures incidence from the treatment-initiation decision point regardless of subsequent continuation. Direct quantitative comparison across these different estimands is therefore not fully appropriate. Balanced consideration of both findings from the two retrospective cohort studies therefore favors cautious clinical interpretation: Neither our finding nor the divergent Lin et al. finding (16), on its own, supports a definitive causal claim in either direction. Even the most rigorous observational design cannot fully resolve unmeasured confounding, and Mendelian randomization approaches capture lifetime genetic risk rather than real-world disease trajectories (29, 30). Definitive resolution requires prospective cohort data with structured psoriasis outcome ascertainment.

Taking the retrospective nature of this study into account, several clinical implications merit consideration. First, AD was associated with an increased risk of subsequent psoriasis. However, given the low absolute incidence of 0.31% over approximately 7 years, these findings do not support routine screening for psoriasis in all patients with AD. Rather, they support clinical awareness that psoriasis may emerge in a subset of patients over time—particularly those with moderate to severe AD. Second, biologic treatment was not associated with increased psoriasis risk. Conversely, it was associated with a lower risk than cvIS. These data may inform treatment counselling and provide reassurance regarding the psoriasis risk profile of type 2-targeted biologics.

## Data Availability

The original contributions presented in the study are included in the article/**Supplementary Material**. Further inquiries can be directed to the corresponding authors.
